# Supplementary material for: Functional Activity in the Effect of Transcranial Magnetic Stimulation Therapy for Patients with Depression: A Meta-Analysis
Source: J Pers Med. 2023 Feb 24;13(3):405. doi: 10.3390/jpm13030405 (PMC10051603; doi:10.3390/jpm13030405)
Supplement: Supplementary file 1 [file jpm-13-00405-s001.zip › jpm-2192866-supplementary.pdf]

Table S1. TMS details of included studies.

Year	Author	Type	Target	Frequency	Intensity	Number of Trains and Durations	Intertrain interval	Pulse per secession	Session frequency	Total sessions
2004	Xingbao Li	rTMS	left DLPFC	100% MT	1Hz	21 trains of 21s	21s	N.A.	N.A.	7
2007	Paul B. Fitzgerald	low-frequency right-sided TMS	right DLPFC	110% MT	1Hz	4 trains of 180 s	30s	12	5 days per week, 10 secessions per day	150
		high-frequency left-sided TMS	left DLPFC	100% MT	10Hz	30 trains of 5 s	25s	160	5 days per week, 10 secessions per day	150
2018	Andrada D. Neacsiu	rTMS	left DLPFC	120% MT	10Hz	75 trains of 4s	26s	3000	5 days per week, 1 secession per day	20
2020	Anhai Zheng	rTMS	left DLPFC	100% MT	10Hz	50 trains of 20 min	21s	1500	five sessions per week	10
2022	Zhou Biao	rTMS	left DLPFC	100% MT	20Hz	50 trains of 2s	28s	2000	five sessions per week	15

DLPFC, dorsolateral prefrontal cortex; MT, motor threshold.

Table S2. MRI details of included studies.

Year	Author	Software	Imaging modalities	Statistical threshold	Correction step	Coordinates space	Task
2004	Xingbao Li	Sun workstations	1.5-T MRI	P<0.05	cluster statistical weight	Talairach	None
2007	Paul B. Fitzgerald	fixed response boxcar function	1.5-T MRI	P<0.05	uncorrected	Talairach	the Tower of London task
2018	Andrada D. Neacsiu	FSL	3-T MRI	P<0.005	voxel-level correction	MNI	The goal priming task
2020	Anhai Zheng	SPM8	3-T MRI	P<0.05	cluster-level correction	MNI	None
2022	Zhou Biao	DPARSF	3-T MRI	p<0.05	uncorrected	MNI	None

MNI, Montreal Neurological Institute space; MRI, magnetic resonance imaging.
